# Associations of folate intake with all-cause and cause-specific mortality among individuals with diabetes

**DOI:** 10.3389/fnut.2022.1021709

**Published:** 2022-10-14

**Authors:** Hexin Su, Yacong Bo, Xiaoan Zhang, Junxi Zhang, Zhan Gao, Zengli Yu

**Affiliations:** ^1^The Fifth Affiliated Hospital of Zhengzhou University, Zhengzhou, China; ^2^School of Public Health, Zhengzhou University, Zhengzhou, China; ^3^The Third Hospital of Zhengzhou University, Zhengzhou, China; ^4^NHC Key Laboratory of Birth Defects Prevention, Henan Key Laboratory of Population Defects Prevention, Zhengzhou, China

**Keywords:** diet, folate, diabetes, mortality, cohort

## Abstract

**Background and aims:**

The evidence regarding folate intake and mortality risk among patients with type 2 diabetes (T2D) remains unclear. This study aimed to investigate the association of folate intake with the risk of mortality among individuals with T2D.

**Methods:**

A total of 9,196 participants with T2D from the Third National Health and Nutrition Examination Survey (NHANES III) and NHANES 1999–2014 were included. The data of survival were obtained by the cohort database linked with the national death index up to 31 December 2015. The Cox proportional hazard model was used to evaluate the relationship between dietary folate with all-cause and cause-specific mortality.

**Results:**

Among patients with T2D, dietary folate intake was negatively correlated with all-cause mortality, cardiovascular mortality, and cancer mortality in men, and for women with all-cause mortality and cardiovascular mortality. The multivariate adjustment hazard ratio (*HR*) (95% *CIs*) for men of highest *vs*. lowest quartile was 0.77 (0.66–0.90), 0.61 (0.45–0.83), and 0.70 (0.49–0.99) for all-cause, cardiovascular, and cancer mortality, respectively. Among women, the multivariate adjustment *HR* (95% *CIs*) of highest *vs*. lowest quartile was 0.77 (0.64–0.92), 0.52 (0.33–0.83), and 0.78 (0.50–1.22) for all-cause, cardiovascular, and cancer mortality, respectively.

**Conclusion:**

Higher dietary intake of folate was significantly associated with lower all-cause and cardiovascular mortality. This cohort study suggested that increasing the dietary folate intake may reduce mortality risk among U.S. adults with T2D.

## Introduction

Folate and its natural derivatives are vitamin B9-based compounds, which are essential micronutrients and are essential for normal cell function. Mammals do not have the ability to synthesize folate and must absorb folate from their diet (1).

Diabetes is one of the most common chronic ailments and a major healthcare problem in worldwide (2). Compared with healthy individuals, previous studies found that patients with T2D had significantly lower circulating folate concentrations (3). And folate has a considerable positive impact on Metabolic syndrome (4), especially diabetes (5, 6). Therefore, it is important to investigate the association of folate with long-term health outcomes in patients with T2D.

Several epidemiological studies have investigated the relationship between dietary folate intake and the risk of death (7–10). Nevertheless, most of these studies were conducted among general population or specific population, such as the elderly and cancer patients. The evidence for patients with diabetes is limited (11–14). Therefore, prospective cohort studies are needed to provide reliable estimates and accurate results for patients with diabetes. To address these research gaps, we prospectively investigated the relationship between folate dietary intake and the risk of all-cause and cause-specific mortality among U.S. patients with T2D, by using national representative sample data from the National Health and Nutrition Examination Survey (NHANES).

## Materials and methods

### Study population

The NHANES is conducted by the National Center for Health Statistics of the Centers for Disease Control and Prevention (CDC). It is a stratified, multistage, and national survey which uses a representative sample of the non-institutionalized civilian population of the U.S.A. (15). Information on lifestyle factors, health, and dietary folate intake status was collected from each participant. In this research, the data of NHANES III (1988–1994) and NHANES from 1999 to 2014 were used. Participants (aged ≥ 20 years old) with T2D were included. T2D was defined by fasting plasma glucose ≥7.0 mmol/L, and/or hemoglobin HbA1c level >6.5%, and/or self-reported doctor-diagnosed T2D, and/or use of insulin or oral hypoglycemic medication. A total of 9,196 patients with diabetes were included. All participants have received written informed consent.

### Measurement of dietary folate intake

Dietary folate data were evaluated by trained interviewers through 24-h dietary recall. During the NHANES III and 1999–2002 survey cycles, participants conducted one single day 24-h dietary recall at the Mobile testing Center (MEC). Since 2003, two dietary recalls have been conducted. The first dietary recall was conducted in-person in MEC, and the second dietary recall was conducted 3–10 days later *via* telephone. Dietary folate intake was assessed using the U.S. Department of Agriculture (USDA) Food and Nutrient Database for Dietary Studies (FNDDS) version 1.0–5.0 (16).

### Ascertainment of mortality

Information on all-cause and cause-specific mortality status was obtained by linking the cohort database with the national death index up to 31 December 2015. The outcomes of the current study were all-cause mortality, cardiovascular mortality, and cancer mortality, which were determined according to the 10th revision of the International Classification of Diseases (ICD-10). Cardiovascular mortality was defined as the ICD-10 code of I00-I09, I11, I13, I20-I51, or I60-I69; and cancer mortality was defined as the ICD-10 code of C00-C97. Each individual's Time-to-event was calculated from the date of recruitment to the date of death or the censor date (31 December 2015), whichever came earlier.

### Covariates

The following variables were considered as covariates based on the literature review: age (years), gender (male or female), race (Hispanic, non-Hispanic white, non-Hispanic black, and racial including multi-race), smoking (never, former, or current), alcohol drinking (never, low to moderate, or heavy), the ratio of family income to poverty (≤1, 1–3, or >3), leisure time physical activity [defined as the product of metabolic equivalent value (MET)] and self-reported cardiovascular disease (yes or no) and hypertension (yes or no). Body mass index (BMI) was calculated as weight (kg) divided by height (meters squared).

### Statistical analysis

We used numbers (percentages) to describe classified variables and means (standard deviation) to describe continuous variables (17). Cox proportional hazard model was used to evaluate the relationship between dietary folate and all-cause and cause-specific mortality. Dietary folate intake was divided into four quartiles; we selected the first quartile as the reference group, and adopt two different models: not adjusted any variable with the crude model and the multivariate model adjusted for age, leisure time physical activity, ethnicity, BMI, family income, smoking, drinking, hypertension, and cardiovascular disease. Stratified analyses were conducted based on age (<60 years old or ≥60 years old), current smoker (yes or no), current drinker (yes or no), and body mass index (<30 or ≥30 kg/m^2^). We examined each potential correction factor separately by adding a multiplicative interaction term (i.e., continuous dietary nutrient intake parameter ^*^ potential correction factor).

To examine the relationships' robustness, we conducted two sensitivity analyses: (1) excluding participants without cardiovascular disease at baseline and (2) excluding patients who died within 2 years of follow-up. All statistical analyses were conducted using SPSS25.0. The *p* < 0.05 was considered to be statistically significant.

## Results

### General characteristics

The general characteristics of the participants are shown in Table 1. A total of 9,196 participants with T2D was included, with an average age of 61.7 (SD: 14.0) years at baseline, 4,507 men (49.01%) and 4,689 (50.99%) women. During a median follow-up of 7.3 years [interquartile range (*IQR*): 3.8–12.4], 3,495 participants died, including 818 from cardiovascular disease and 607 from cancer. Compared with survivors, participants who died were more likely to be elderly, female, and non-Hispanic white; less likely to be never smoker; and more likely to have hypertension and cardiovascular disease at baseline.

**Table 1:** Baseline characteristics of the study population.

**Variables**	**Total sample**	**Survivors**	**Death**
	***N*** = **9,196**	***N*** = **5,701 (61.99%)**	***N*** = **3,495 (38.01%)**
Age, year*	61.7 (14.0)	57.6 (13.7)	68.3 (11.7)
**Sex#**			
Female	4,689 (50.99%)	2,993 (52.50%)	1,696 (48.53%)
Male	4,507 (49.01%)	2,708 (47.50%)	1,799 (51.47%)
**Race#**			
Hispanic	2,678 (29.12%)	1,813 (31.80%)	865 (24.75%)
Non-Hispanic White	3,538 (38.47%)	1,913 (33.56%)	1,625 (46.49%)
Non-Hispanic Black	2,422 (26.34%)	1,520 (26.66%)	902 (25.81%)
Other	558 (6.07%)	455 (7.98%)	103 (2.95%)
**Drink#**			
Never	3,834 (41.69%)	2,084 (36.55%)	1,750 (50.07%)
Low to moderate	2,077 (22.59%)	1,292 (22.66%)	785 (22.46%)
Heavy	3,285 (35.72%)	2,325 (40.78%)	960 (27.47%)
**Smoke#**			
Never	4,407 (47.92%)	2,933 (51.45%)	1,474 (42.17%)
Former	3,208 (34.88%)	1,793 (31.45%)	1,415 (40.49%)
Current	1,581 (17.19%)	975 (17.10%)	606 (17.34%)
**Income#**			
<1	2,284 (23.98%)	1,367 (26.24%)	917 (24.84%)
1~3	4,422 (45.22%)	2,578 (52.76%)	1,844 (48.09%)
>3	2,490 (30.80%)	1,756 (21.00%)	734 (27.08%)
Hypertension#	2,776 (30.19%)	1,364 (23.93%)	1,412 (40.4%)
Cardiovascular disease#	1,549 (16.84%)	731 (12.82%)	818 (23.40%)
BMI, kg/m^2*^	30.77 (6.28)	31.61 (6.28)	29.40 (6.05)
Leisure-time physical activity, MET* MET	14.49 (12.20)	14.31 (12.06)	14.79 (12.41)

^*^Data were presented as means (standard deviations).

# Data were presented as numbers (percentages).

BMI, body mass index; MET, metabolic equivalent value.

### Dietary folate with all-cause and cause-specific mortality

The sex-specific relationship between dietary folate intake and all-cause and cause-specific mortality among patients with T2D is shown in Table 2. In men, we found that folate intake was negatively correlated with all-cause mortality, cardiovascular mortality, and cancer mortality. The multivariate adjustment hazard ratio (*HR*) (95% *CIs*) of men highest *vs*. lowest quartile was 0.77 (0.66–0.90), 0.61 (0.45–0.83), and 0.70 (0.49–0.99) for all-cause mortality, cardiovascular mortality, and cancer mortality, respectively. In women, folate intake was negatively correlated with all-cause mortality and cardiovascular mortality, but not with cancer mortality. The highest *vs*. lowest quartile HR (95% *CIs*) was 0.77 (0.64–0.92), 0.52 (0.33–0.83), and 0.78 (0.50–1.22) for all-cause, cardiovascular, and cancer mortality, respectively.

**Table 2:** Hazard ratio (*HRs*) and 95% *CIs* for all-cause and cause-specific mortality according to Quintiles of dietary Folate.

	**Nutrient intake**				**P_trend_**
	**Q1**	**Q2**	**Q3**	**Q4**	
**Male**
**All-cause mortality**				
Crude model	Ref	0.93 (0.82~1.05)	0.89 (0.77~1.02)	0.68 (0.5~0.79)	<0.001
Multivariable model	Ref	0.93 (0.82~1.05)	0.93 (0.81~1.07)	0.77 (0.66~0.90)	0.009
**Cardiovascular mortality**			
Crude model	Ref	0.95 (0.75~1.21)	0.77 (0.58~1.02)	0.59 (0.43~0.79)	0.003
Multivariable model	Ref	0.90 (0.71~1.15)	0.76 (0.57~1.01)	0.61 (0.45~0.83)	0.012
**Cancer mortality**				
Crude model	Ref	0.68(0.51~0.91)	0.81 (0.60~1.10)	0.54 (0.39~0.76)	0.002
Multivariable model	Ref	0.70 (0.52~0.94)	0.93 (0.68~1.26)	0.70 (0.49~0.99)	0.049
**Female**
**All-cause mortality**				
Crude model	Ref	0.95 (0.84~1.07)	0.92 (0.80~1.06)	0.74 (0.62~0.89)	0.014
Multivariable model	Ref	0.89 (0.80~1.01)	0.86 (0.74–0.99)	0.77 (0.64~0.92)	0.016
**Cardiovascular mortality**			
Crude model	Ref	0.97 (0.74~1.25)	0.84 (0.61~1.14)	0.48 (0.30~0.77)	0.018
Multivariable model	Ref	0.91 (0.70~1.18)	0.78 (0.57~1.08)	0.52 (0.33~0.83)	0.037
**Cancer mortality**			
Crude model	Ref	0.87 (0.64~1.19)	1.01 (0.72~1.42)	0.76 (0.49~1.18)	0.532
Multivariable model	Ref	0.83 (0.61~1.13)	1.02 (0.72~1.43)	0.78 (0.50~1.22)	0.461

Crude HR: did not adjust anything. Multivariable HR: adjusted for age, race/ethnicity, BMI, family income-poverty ratio, smoking, drinking, leisure-time physical activity, hypertension, and cardiovascular disease.

### Stratified analyses and sensitivity analysis

The relationships between dietary folate intake and all-cause and cause-specific mortality were generally similar among subgroups, although some differences were not statistically significant, as shown in Table 3. A statistically significant interaction was observed for some factors. We observed that folate intake had a stronger negative correlation with all-cause and cause-specific mortality in the obese men. For female participants, folate intake was negatively correlated with all-cause mortality and cause-specific mortality of recent smoking and drinking patients. Sensitivity analysis showed that the excluding of participants died within 2 years of follow-up (Table 4) or excluding participants who had cardiovascular disease at baseline (Table 5), the significant association between dietary folate and the risk of mortality was still similar.

**Table 3:** Stratified analyses of the associations between dietary folate intake with all-cause and cause-specific mortality.

	**Nutrient intake**				**P_trend_**	**P_interaction_**
	**Q1**	**Q2**	**Q3**	**Q4**		
**Male**					
**All-cause mortality**						
Age						0.065
<60 y	Ref	1.30 (0.99~1.72)	1.42 (1.03~1.96)	1.12 (0.79~1.60)	0.106	
≥60 y	Ref	0.85 (0.74~0.98)	0.84 (0.72~0.99)	0.69 (0.59~0.82)	<0.001	
Current smoker					0.606
Yes	Ref	1.01 (0.77~1.34)	1.18 (0.87~1.60)	0.77 (0.53~1.10)	0.149	
No	Ref	0.91 (0.79~1.04)	0.87 (0.75~1.02)	0.75 (0.64~0.89)	0.010	
Current drinker					0.976
Yes	Ref	0.92 (0.73~1.16)	0.88 (0.67~1.15)	0.77 (0.57~1.03)	0.358	
No	Ref	0.93 (0.81~1.08)	0.95 (0.81~1.12)	0.76 (0.63~0.90)	0.018	
BMI						0.033
<30 kg/m^2^	Ref	0.91 (0.78~1.07)	0.97 (0.82~1.16)	0.78 (0.64~0.94)	0.053	
≥30 kg/m^2^	Ref	0.93 (0.76~1.15)	0.86 (0.68~1.08)	0.73 (0.57~0.94)	0.095	
**Cardiovascular mortality**						
**Age**						0.237
<60 y	Ref	1.47 (0.84~2.56)	1.27 (0.66~2.45)	0.76 (0.34~1.71)	0.294	
≥60 y	Ref	0.86 (0.65~1.13)	0.72 (0.52~0.98)	0.63 (0.45~0.88)	0.034	
**Current smoker**					0.375
Yes	Ref	1.23 (0.66~2.30)	1.34 (0.69~2.61)	0.98 (0.47~2.08)	0.755	
No	Ref	0.89 (0.68~1.16)	0.70 (0.51~0.97)	0.59 (0.42~0.83)	0.012	
**Current drinker**					0.930
Yes	Ref	0.77 (0.48~1.22)	0.80 (0.48~1.36)	0.65 (0.37~1.16)	0.482	
No	Ref	0.99 (0.74~1.33)	0.77 (0.55~1.08)	0.63 (0.44~0.91)	0.040	
**BMI**						0.190
<30 kg/m^2^	Ref	0.86 (0.63~1.17)	0.85 (0.59~1.21)	0.65 (0.44~0.96)	0.195	
≥30 kg/m^2^	Ref	1.04 (0.70~1.56)	0.73 (0.45~1.17)	0.66 (0.39~1.10)	0.181	
**Cancer mortality**						
Age						0.394
<60 y	Ref	1.28 (0.65~2.51)	2.26 (1.11~4.60)	1.12 (0.47~2.66)	0.116	
≥60 y	Ref	0.60 (0.43~0.83)	0.73 (0.51~1.03)	0.58 (0.40~0.85)	0.005	
**Current smoker**					0.522
Yes	Ref	0.68 (0.36~1.30)	1.21 (0.65~2.25)	1.05 (0.53~2.09)	0.439	
No	Ref	0.67 (0.48~0.93)	0.79 (0.55~1.12)	0.54 (0.36~0.82)	0.014	
**Current drinker**					0.423
Yes	Ref	0.60 (0.35~1.04)	0.82 (0.45~1.50)	0.68 (0.35~1.34)	0.307	
No	Ref	0.74 (0.52~1.06)	0.94 (0.65~1.35)	0.66 (0.44~0.99)	0.132	
**BMI**						0.883
<30 kg/m^2^	Ref	0.72 (0.51~1.04)	0.78 (0.52~1.17)	0.61 (0.39~0.95)	0.114	
≥30 kg/m^2^	Ref	0.62 (0.37~1.05)	1.04 (0.63~1.72)	0.67 (0.38~1.18)	0.117	
**Female**						
**All-cause mortality**						
**Age**						0.303
<60 y	Ref	0.93 (0.70~1.24)	0.78 (0.54~1.13)	1.14 (0.75~1.74)	0.434	
≥60 y	Ref	0.89 (0.78~1.02)	0.85 (0.73~1.00)	0.73 (0.59~0.89)	0.012	
**Current smoker**					0.001
Yes	Ref	0.85 (0.62~1.15)	0.53 (0.33~0.85)	0.55 (0.32~0.94)	0.019	
No	Ref	0.91 (0.80~1.05)	0.91 (0.79~1.06)	0.85 (0.70~1.03)	0.286	
**Current drinker**					0.006
Yes	Ref	0.79 (0.63~1.00)	0.65 (0.49~0.85)	0.66 (0.47~0.92)	0.006	
No	Ref	0.94 (0.81~1.08)	0.97 (0.82~1.14)	0.86 (0.69~1.08)	0.569	
**BMI**						0.454
<30 kg/m^2^	Ref	0.81 (0.69~0.95)	0.83 (0.68~1.00)	0.82 (0.65~1.04)	0.041	
≥30 kg/m^2^	Ref	1.03 (0.86~1.24)	0.93 (0.75~1.16)	0.77 (0.58~1.03)	0.261	
**Cardiovascular mortality**						
**Age**						0.215
<60 y	Ref	1.07 (0.54~2.13)	0.62 (0.23~1.67)	1.19 (0.42~3.34)	0.737	
≥60 y	Ref	0.90 (0.68~1.20)	0.79 (0.57~1.11)	0.45 (0.27~0.76)	0.025	
**Current smoker**					0.453
Yes	Ref	0.68 (0.31~1.49)	0.80 (0.30~2.13)	0.38 (0.08~1.68)	0.544	
No	Ref	0.98 (0.74~1.29)	0.80 (0.57~1.12)	0.57 (0.35~0.92)	0.095	
**Current drinker**					0.163
Yes	Ref	1.18 (0.71~1.96)	0.85 (0.46~1.58)	0.37 (0.13~1.06)	0.171	
No	Ref	0.83 (0.61~1.13)	0.77 (0.53~1.12)	0.62 (0.37~1.05)	0.210	
**BMI**						0.218
<30 kg/m^2^	Ref	0.85 (0.60~1.22)	0.85 (0.56~1.28)	0.62 (0.35~1.10)	0.395	
≥30 kg/m^2^	Ref	1.04 (0.70~1.53)	0.75 (0.45~1.24)	0.46 (0.21~1.02)	0.168	
**Cancer mortality**						
**Age**						0.497
<60 y	Ref	0.56 (0.30~1.05)	0.60 (0.29~1.25)	0.63 (0.26~1.53)	0.207	
≥60 y	Ref	0.98 (0.68~1.42)	1.17 (0.79~1.73)	0.85 (0.51~1.41)	0.673	
**Current smoker**					0.011
Yes	Ref	0.67 (0.37~1.24)	0.44 (0.17~1.16)	0.44 (0.15~1.28)	0.176	
No	Ref	0.91 (0.63~1.30)	1.20 (0.82~1.74)	0.92 (0.57~1.50)	0.560	
**Current drinker**					0.022
Yes	Ref	0.67 (0.41~1.10)	0.39 (0.20~0.76)	0.61 (0.31~1.17)	0.034	
No	Ref	0.93 (0.62~1.39)	1.59 (1.07~2.36)	0.95 (0.52~1.70)	0.061	
**BMI**						0.131
<30 kg/m^2^	Ref	0.69 (0.44~1.09)	1.19 (0.74~1.90)	1.02 (0.58~1.81)	0.226	
≥30 kg/m^2^	Ref	0.99 (0.64~1.52)	0.89 (0.55~1.47)	0.59 (0.29~1.18)	0.492	

Adjusted for age, race/ethnicity, BMI, leisure-time physical activity, family income-poverty ratio, smoking (not for smoke stratified analysis), drinking (not for drinking stratified analysis), hypertension, and cardiovascular disease.

**Table 4:** Hazard ratio and 95% *CIs* for all-cause and cause-specific mortality according to quartiles of dietary folate intake after excluding participants with cardiovascular disease at baseline.

	**Nutrient intake**				**P_trend_**
	**Q1**	**Q2**	**Q3**	**Q4**	
**Male**
All-cause mortality	Ref	0.91 (0.79~1.05)	0.93 (0.79~1.09)	0.76 (0.64~0.92)	0.034
Cardiovascular mortality	Ref	0.87 (0.64~1.17)	0.77 (0.54~1.10)	0.70 (0.48~1.03)	0.253
Cancer mortality	Ref	0.69 (0.50~0.96)	0.94 (0.67~1.32)	0.69 (0.47~1.01)	0.069
**Female**
All-cause mortality	Ref	0.93 (0.81~1.06)	0.81 (0.69~0.96)	0.77 (0.62~0.95)	0.019
Cardiovascular mortality	Ref	0.96 (0.71~1.29)	0.79 (0.54~1.15)	0.55 (0.32~0.95)	0.139
Cancer mortality	Ref	0.85 (0.61~1.18)	1.02 (0.71~1.47)	0.77 (0.48~1.24)	0.556

Adjusted for age, race/ethnicity, BMI, family income-poverty ratio, smoking, drinking, leisure-time physical activity, and hypertension.

**Table 5:** Hazard ratios and 95% *CIs* for all-cause and cause-specific mortality according to quartiles of dietary folate intake after excluding participants who were died within 2 years of follow-up.

	**Nutrient intake**				**P_trend_**
	**Q1**	**Q2**	**Q3**	**Q4**	
**Male**
All-cause mortality	Ref	0.97 (0.85~1.11)	1.02 (0.88~1.19)	0.82 (0.70~0.97)	0.065
Cardiovascular mortality	Ref	0.96 (0.73~1.25)	0.87 (0.63~1.19)	0.72 (0.51~1.01)	0.272
Cancer mortality	Ref	0.69 (0.50~0.95)	0.89 (0.64~1.25)	0.63 (0.43~0.93)	0.038
**Female**
All-cause mortality	Ref	0.94 (0.82~1.06)	0.84 (0.72~0.98)	0.81 (0.66~0.99)	0.063
Cardiovascular mortality	Ref	0.98 (0.74~1.29)	0.77 (0.54~1.10)	0.52 (0.31~0.88)	0.060
Cancer mortality	Ref	0.88 (0.63~1.22)	0.86 (0.58~1.27)	0.93 (0.59~1.48)	0.821

Adjusted for age, race/ethnicity, BMI, family income-poverty ratio, smoking status, drinking, leisure-time physical activity, hypertension, and cardiovascular disease.

## Discussion

Our study has shown that, among patients with T2D, dietary folate intake was negatively correlated with all-cause, cardiovascular, and cancer mortality in men, and negatively correlated with all-cause and cardiovascular mortality in women. And the excluding of participants who were died within 2 years of follow-up or those who had cardiovascular disease at baseline, the conclusion did not change. The association might because of glucose homeostasis (18), vascular protection (19, 20), or homocysteine methylation (21, 22) which benefit from dietary folate.

This reverse relationship is consistent with findings from previous studies. A non-linear correlation was found between serum folate levels with CVD morbidity and all-cause mortality in 7,700 American adults with T2D (23). The Swedish cohort study showed that dietary folate intake was negatively correlated with the risk of all-cause mortality among women who were diagnosed with breast cancer (24). A Collaborative Cohort study from Japan found that dietary folate intake was negatively correlated with heart failure mortality in men and coronary heart disease mortality and total cardiovascular mortality in women (25). A Spanish ecological study showed that folate intake was inversely associated with coronary artery disease mortality and cerebrovascular disease mortality in men and cerebrovascular disease mortality in women, and the protective effect is positively correlated with folate intake (26). However, no previous studies investigated the relationship of dietary folate intake with mortality among patients with T2D. In the present study, we found a negative relationship between folate intake and cardiovascular and all-cause mortality among 9,196 U.S. adults with T2D, which provided further evidence for the prevention of premature death in this specific population.

However, it is inconsistent with the results of some related studies in the U.S.A. A study by the American Association of Retirement Diet and Health found no significant relationship between folate intake and liver disease mortality (27). Another study suggested that dietary folate was not associated with breast cancer-specific mortality or all-cause mortality (28). Other studies have shown that serum folate levels are not associated with CVD mortality in participants with T2D (29). These inconsistencies may be caused by series of factors, which include differences of research methods and inadequate data collection, as well as differences in the populations or health outcomes.

In this study, a representative American population was obtained by using a complex, stratified, and multi-stage probability sampling method. The large sample size enabled us to obtain relatively robust and accurate estimates. In addition, based on the comprehensive data collected by NHANES, a wide range of potential confounding factors was adjusted.

Our research also had several limitations. First, dietary folate intake was collected through one or two 24-h dietary recalls at a single point, which may not well-reflect long-term intake because of large changes in daily food intake. In addition, the information of folate intake was evaluated by questionnaire, which may bring recall bias. However, this measurement bias seems to be random, and there is no evidence suggesting that the potential measurement bias is different between the participants who survived and those who died. We thus speculate that this limitation should not affect the study's conclusion. Second, most diabetic patients received different diets and drugs because of their symptoms. Metformin, as the first choice for diabetes, may significantly reduce folate absorption, and critically ill patients may have more folate intake and higher mortality, so the possibility of “causality inversion” can't be ruled out. Third, the level of folate intake varied between different studies, which make it difficult to compare our results with other studies directly. In addition, residues or unknown mixtures cannot be completely excluded.

## Conclusion

In conclusion, using the data NHANES, we found that dietary folate intake was negatively correlated with all-cause mortality, cardiovascular mortality, and cancer mortality in men and with all-cause mortality and cardiovascular mortality in women who were diagnosed with T2D. This study suggested the increasing of intake of dietary folate may reduce the risk of mortality in U.S. adults with T2D.

## Data availability statement

Publicly available datasets were analyzed in this study. This data can be found at: https://wwwn.cdc.gov/nchs/nhanes/Default.aspx.

## Ethics statement

Ethical review and approval was not required for the study on human participants in accordance with the local legislation and institutional requirements. The patients/participants provided their written informed consent to participate in this study.

## Author contributions

YB and ZY: conceptualization and supervision. ZG and JZ: methodology. HS: data curation and writing—original draft preparation. HS, YB, and ZY: writing—review and editing. ZY: project administration. All authors contributed to the article and approved the submitted version.

## Funding

This work was supported by Special Major Public Welfare Project of Henan Province No. 201300310800 and Open Research Fund of National Health Commission Key Laboratory of Birth Defects Prevention and Henan Key Laboratory of Population Defects Prevention No. ZD202203.

## Conflict of interest

The authors declare that the research was conducted in the absence of any commercial or financial relationships that could be construed as a potential conflict of interest.

## Publisher's note

All claims expressed in this article are solely those of the authors and do not necessarily represent those of their affiliated organizations, or those of the publisher, the editors and the reviewers. Any product that may be evaluated in this article, or claim that may be made by its manufacturer, is not guaranteed or endorsed by the publisher.

## References

[1] Toit AID. Synthetic folic acid - food folate and 5-mthf are not the same thing. Medical Chronicle. (2019 December, 30). Available online at: https://hdl.handle.net/10520/EJC-1370319f85

[2] Legenbauer T Benecke A Beutel ME. Diabetes Mellitus und Essstörungen: Herausforderungen für die interdisziplinäre Behandlung. Berlin, Boston, MA: De Gruyter (2021). 10.1515/9783110583205

[3] Malaguarnera G Gagliano C Salomone S Giordano M Bucolo C Pappalardo A. Folate status in type 2 diabetic patients with and without retinopathy. Clin Ophthalmol. (2015) 9:1437–42. 10.2147/OPTH.S77538 26300625 PMC4536839

[4] Ashok T Puttam H Tarnate VCA Jhaveri S Avanthika C Trejo Treviño AG. Role of vitamin B12 and folate in metabolic syndrome. Cureus. (2021) 13:e18521. 10.7759/cureus.18521 34754676 PMC8569690

[5] Gargari BP Aghamohammadi V Aliasgharzadeh A. Effect of folic acid supplementation on biochemical indices in overweight and obese men with type 2 diabetes. Diabetes Res Clin Pract. (2011) 94:33–8. 10.1016/j.diabres.2011.07.003 21802161

[6] Asbaghi O Ashtary-Larky D Bagheri R Moosavian SP Olyaei HP Nazarian B. Folic acid supplementation improves glycemic control for diabetes prevention and management: a systematic review and dose-response meta-analysis of randomized controlled trials. Nutrients. (2021) 13:2355. 10.3390/nu13072355 34371867 PMC8308657

[7] Danaei G Ding EL Mozaffarian D Taylor B Rehm J Murray CJ. The preventable causes of death in the United States: comparative risk assessment of dietary, lifestyle, and metabolic risk factors. PLoS Med. (2009) 6:e1000058. 10.1371/journal.pmed.1000058 19399161 PMC2667673

[8] Zhu J Chen C Lu L Yang K Reis J He K. Intakes of folate, vitamin B(6), and vitamin B(12) in relation to diabetes incidence among American young adults: a 30-year follow-up study. Diabetes Care. (2020) 43:2426–34. 10.2337/dc20-0828 32737139 PMC7510025

[9] Xu X Wei W Jiang W Song Q Chen Y Li Y. Association of folate intake with cardiovascular-disease mortality and all-cause mortality among people at high risk of cardiovascular-disease. Clin Nutr. (2022) 41:246–54. 10.1016/j.clnu.2021.11.007 34929527

[10] Bo Y Xu H Zhang H Zhang J Wan Z Zhao X. Intakes of folate, vitamin B6, and vitamin B12 in relation to all-cause and cause-specific mortality: a national population-based cohort. Nutrients. (2022) 14:2253. 10.3390/nu14112253 35684053 PMC9182598

[11] El-Khodary NM Dabees H Werida RH. Folic acid effect on homocysteine, Sortilin levels and glycemic control in type 2 diabetes mellitus patients. Nutr Diabetes. (2022) 12:33. 10.1038/s41387-022-00210-6 35732620 PMC9217798

[12] Li K Chen X Hu Y Gao B. Folic acid supplementation diminishes diabetes induced neural tube defects by recovering impaired embryo gene expression through its antioxidant activity. J Pharm Res Int. (2021) 33:258–71. 10.9734/jpri/2021/v33i62A3520435024509

[13] Hayden MR Tyagi SC. Impaired folate-mediated one-carbon metabolism in type 2 diabetes, late-onset Alzheimer's disease and long COVID. Medicina. (2021) 58:16. 10.3390/medicina58010016 35056324 PMC8779539

[14] Keerti A Jankar J. Role of folic acid in type 2 diabetes. J Pharm Res Int. (2021) 33:137–43. 10.9734/jpri/2021/v33i60A3446535024509

[15] Archer E Pavela G Lavie CJ. The inadmissibility of what we eat in America and Nhanes dietary data in nutrition and obesity research and the scientific formulation of national dietary guidelines. Mayo Clin Proc. (2015) 90:911–26. 10.1016/j.mayocp.2015.04.009 26071068 PMC4527547

[16] U.S. Department of Agriculture Food Surveys Research Group. Food and Nutrient Database for Dietary Studies. Beltsville, Md. Available online at: Https://Www.Ars.Usda.Gov/Northeast-Area/Beltsville-Md-Bhnrc/Beltsville-Human-Nutrition-Research-Center/Food-Surveys-Research-Center/Food-Surveys-Research-Group/Docs/Fndds/ (accessed December 2, 2021).

[17] Zhang Z. Multiple imputation with multivariate imputation by chained equation (mice) package. Ann Transl Med. (2016) 4:30. 10.3978/j.issn.2305-5839.2015.12.63 26889483 PMC4731595

[18] Bidhendi Yarandi R. Effect of folate supplementation on insulin sensitivity and type 2 diabetes: a meta-analysis of randomized controlled trials. Am J Clin Nutr. (2019) 109:1233. 10.1093/ajcn/nqz021 30949668

[19] Boykin JV Jr Hoke GD Driscoll CR Dharmaraj BS. High-dose folic acid and its effect on early stage diabetic foot ulcer wound healing. Wound Repair Regen. (2020) 28:517–25. 10.1111/wrr.12804 32141182

[20] Wang Z Xing W Song Y Li H Liu Y Wang Y. Folic acid has a protective effect on retinal vascular endothelial cells against high glucose. Molecules. (2018) 23:2326. 10.3390/molecules23092326 30213067 PMC6225375

[21] Muzurović E Kraljević I Solak M Dragnić S Mikhailidis DP. Homocysteine and diabetes: role in macrovascular and microvascular complications. J Diabetes Complications. (2021) 35:107834. 10.1016/j.jdiacomp.2020.107834 33419630

[22] Mursleen MT Riaz S. Implication of homocysteine in diabetes and impact of folate and vitamin B12 in diabetic population. Diabetes Metab Syndr. (2017) 11(Suppl. 1):S141–6. 10.1016/j.dsx.2016.12.023 28024829

[23] Liu Y Geng T Wan Z Lu Q Zhang X Qiu Z. Associations of serum folate and vitamin B12 levels with cardiovascular disease mortality among patients with type 2 diabetes. JAMA Netw Open. (2022) 5:e2146124. 10.1001/jamanetworkopen.2021.46124 35099545 PMC8804919

[24] Harris HR Bergkvist L Wolk A. Folate intake and breast cancer mortality in a cohort of swedish women. Breast Cancer Res Treat. (2012) 132:243–50. 10.1007/s10549-011-1838-y 22037788 PMC3747350

[25] Cui R Iso H Date C Kikuchi S Tamakoshi A. Dietary folate and vitamin B6 and B12 intake in relation to mortality from cardiovascular diseases: Japan collaborative cohort study. Stroke. (2010) 41:1285–9. 10.1161/STROKEAHA.110.578906 20395608

[26] Medrano MJ Sierra MJ Almazán J Olalla MT López-Abente G. The association of dietary folate, B6, and B12 with cardiovascular mortality in Spain: an ecological analysis. Am J Public Health. (2000) 90:1636–8. 10.2105/AJPH.90.10.1636 11030004 PMC1446360

[27] Persson EC Schwartz LM Park Y Trabert B Hollenbeck AR Graubard BI. Alcohol consumption, folate intake, hepatocellular carcinoma, and liver disease mortality. Cancer Epidemiol Biomarkers Prev. (2013) 22:415–21. 10.1158/1055-9965.EPI-12-1169 23307533 PMC3596467

[28] Xu X Gammon MD Wetmur JG Bradshaw PT Teitelbaum SL Neugut AI. B-vitamin intake, one-carbon metabolism, and survival in a population-based study of women with breast cancer. Cancer Epidemiol Biomarkers Prev. (2008) 17:2109–16. 10.1158/1055-9965.EPI-07-2900 18708404 PMC2673236

[29] Loria CM Ingram DD Feldman JJ Wright JD Madans JH. Serum folate and cardiovascular disease mortality among us men and women. Arch Intern Med. (2000) 160:3258–62. 10.1001/archinte.160.21.3258 11088087

